# Transcriptome Sequencing Analysis Reveals the Regulation of the Hypopharyngeal Glands in the Honey Bee, *Apis mellifera carnica* Pollmann

**DOI:** 10.1371/journal.pone.0081001

**Published:** 2013-12-10

**Authors:** Zhenguo Liu, Ting Ji, Ling Yin, Jie Shen, Fang Shen, Guohong Chen

**Affiliations:** College of Animal Science and Technology, Yangzhou University, Yangzhou, Jiangsu, China; Goethe University Frankfurt, Germany

## Abstract

Transcriptome sequencing has become the main methodology for analyzing the relationship between genes and characteristics of interests, particularly those associated with diseases and economic traits. Because of its role of functional food for humans, commercial royal jelly (RJ) and its production are major research focuses in the field of apiculture. Multiple lines of evidence have demonstrated that many factors affect RJ output by activating or inhibiting various target genes and signaling pathways. Available coding sequences from the Honey Bee Genome Sequencing Consortium have permitted a pathway-based approach for investigating the development of the hypopharyngeal glands (HGs). In the present study, 3573941, 3562730, 3551541, 3524453, and 3615558 clean reads were obtained from the HGs of five full-sister honey bee samples using Solexa RNA sequencing technology. These reads were then assembled into 18378, 17785, 17065, 17105, and 17995 unigenes, respectively, and aligned to the DFCI Honey Bee Gene Index database. The differentially expressed genes (DEGs) data were also correlated with detailed morphological data for HGs acini.

## Introduction

Due to their role in pollination and the benefits of their products, eusocial honey bees are among the most beneficial insects on earth. Currently, there are only eight recognized species of honey bee, with a total of 44 subspecies [1], [2].

Honeybee worker bees display an extraordinarily elaborate division of labor by age [3] and the dramatical changes of behavior of worker bees were definitely correlated with age polyethism [4]. The hypopharyngeal glands (HGs) of the honey bee are age-dependent structures that change with the size of the acini, which are believed to correspond to various social behaviors [5]. HGs develop particularly in worker bees rather than queen and male bees, and degenerate when the task switched from nursing in hive to foraging in the field approximate 18 days after the eclosion and emergence of new workers [6], [7]. The HGs are a pair of exocrine glands that are symmetrically coiled in the front of the head and consist of approximately1000 oval lobules or acini attached to an axial duct. The apiculture product royal jelly (RJ) is secreted from the HGs. Tables S1 and S2 in Document S1 list the components of fresh RJ, which is a complex mixture of different nutritional ingredients synthesized and produced by the HGs in young nurse bees as food of the larvae and adult queen [8]. As a rich concentrated food, RJ is also marketed as a dietary supplement for humans. The various health benefits associated with RJ have been attributed to its remarkable concentrations of proteins, lipids, carbohydrates, vitamins, enzymes, trace mineral substances, and specific vital factors that act as biocatalysts in cell regeneration in the human body as well as in bees. Because its action seems to be systemic rather than occurring via specific biological process, RJ has been recommended for a variety of purposes.

The overall composition of RJ is 67% water; 12.5% crude protein, including small amounts of different amino acids; 11% simple sugars (monosaccharides); and (5%) fatty acids. It also contains trace minerals, enzymes, antibacterial, antibiotic components, and trace amounts of the following vitamins: B1, B2, B6, C, E, [9] niacin (B3), pantothenic acid (B5), biotin (B7), inositol (B8), and folic acid (B9) [10].

The honey bee *Apis mellifera* is an ideal model organism for investigating particular biological phenomena and characteristics, molecular mechanisms and evolution of social behavior [11], [12]. Honey bee studies may, therefore provide insight into related mechanisms in other organisms [13]. The recent publication of the complete *Apis mellifera* genome sequence [14] has provided a foundational resource that is critical for the rapidly growing field of comparative genomics and will accelerate the identification and characterization of genes that modulate behaviors and development [15]. Previous studies have demonstrated that to some extent, the roles of worker bees are flexible, depending on various conditions such as colony demography [7], [16], nutritional status [17], [18], colony conditions [19] and season 20,21. An analysis of differentially expressed genes (DEGs) in the HGs of workers revealed that a buffy homolog and MMP1 (matrix metalloproteinase 1) were differentially expressed in nurse bees and forager, with the tissue-preferential expression reflecting the age-dependent behavioral change in nursing and the later transition to foraging [22]. Ohashi K. et al demonstrated that a 64-kDa protein, RJP57-1, was expressed specifically in the nurse-bee HGs, whereas a 56-kDa protein was expressed in both the nurse-bee and forager-bee [23]. Protein profiling of HGs at different developmental phases were screened by two dimensional electrophoresis methods, and analyzed through network approach to build up 35 key node proteins in the biochemical networks of the HG [24]. However, the secretions produced by the HGs depend on the need [25], such as the RJ components, α-glucosidase [23], glucosidase oxidase [26], galactosidase [27], esterase, lipase and leucine arylamidase [25], [28]were secreted according to the development for the adaptability and preparation for the task switching.

Although improved genetic stocks and good management techniques are the most prominent approaches for increasing the yields of RJ, the molecular mechanisms that underlie HGs development and RJ secretion are not well characterized yet. To investigate the causal relationship between HGs development and RJ secretion, morphological analysis and RNA-seq of HGs dissected from honey bees at different ages were performed. Considerable variations in gene expression were associated with development and metabolism. Thus, a subset of related genes may influence changes in HG development and morphology with age.

## Materials and Methods

### 2.1 Sample Collection

Full-sister honey bees (*Apis mellifera carnica* Pollmann) from the apiaries of Yangzhou University were used throughout the experiment. More than 10 sexually mature virgin queens were artificially fertilized with sperm collected from an isolated, sexually mature drone using an artificial insemination instrument (Apiculture Science Institute of Jilin Province, China) to minimize noise in the genetic background. The best colony in terms of health and fertility was selected for the experiments. Worker bees were marked with paint on the thorax when emerging from the cells. A total of 60 of the marked workers were collected on days 3, 6, 9, 12, and 16 in June 2012. The HGs were dissected for various analyses using a binocular stereomicroscope immediately after anesthetization on ice. Thirty HG heads from each group were infiltrated in 2.5% glutaraldehyde for morphological analysis, and another 30 HGs were frozen in liquid nitrogen for RNA-seq.

### 2.2 RNA Extraction, Library Preparation, and Sequencing

Total RNA was extracted from the HGs of the samples (each pooled from 30 honey bees) using TRIzol reagent (Invitrogen, USA). A Qubit fluorometer (Invitrogen, USA) and an Agilent 2100 Bioanalyzer (Agilent Technologies, Inc., USA) were used to determine the quality and quantity of the RNA [29]. The mRNA was enriched using oligo (dT) magnetic beads, then fragmented into short fragments (approximately 200–700 nt) using fragmentation buffer (Invitrogen, USA). For first-strand cDNA synthesis, the mRNA fragments were used as the template and a random hexamer primer was used according to the manufacturer’s instructions. Following second strand synthesis, the double-stranded cDNA product was purified using the QiaQuick PCR extraction kit (Qiagen, USA) and eluted with EB buffer for end repair and poly(A) addition. Finally, sequencing adapters were ligated to the fragments and the fragments were purified by agarose gel electrophoresis (AGE) and enriched by PCR amplification. The library products were sequenced using the Illumina HiSeq™ 2000 system.

### 2.3 Acquisition of the Raw Data and Statistical Analysis

The original sequence data, or raw data reads, were saved as a FASTQ file, which included the detailed read sequences and quality information. FastQC was used for quality control analysis and to filter out “dirty” raw reads (Figure S1), such as reads with adapters, reads with more than 10% unknown bases, and low quality reads, which were defined as reads having more than 50% bases with a quality value≤5. The clean reads obtained were used for subsequent analyses.

The clean reads were mapped to reference sequences using the SOAP2 alignment algorithm, with a tolerance of no more than two mismatches. The sequencing saturation (Figure S2), distribution of reads (Figure S3), and coverage (Figure S4) were used to assess quality. The unigenes were aligned and mapped to the DFCI Honey Bee Gene Index database, which includes 25007 unigenes, as well to reseq, mRNA, and EST data in GenBank. The reported sequencing data has been approved and assigned to a public data repository (GEO accession number: GSE47136).

### 2.4 Functional Analysis of DEGs

The level of gene expression was calculated as RPKM (reads per kilo base transcriptome per million mapped reads) [30]. This method eliminates the influence of different gene lengths and sequencing discrepancies on the calculation of gene expression and can therefore be used to directly compare DEGs among samples. DEGs were identified using an FDR≤0.001 (false discovery rate no greater than 0.001) [31] and an absolute value of log_2_Ratio≥1 (two-fold change) as the significance thresholds for DGEs. More stringent criteria, with a smaller FDR or greater fold-change value, were used for subsequent analyses.

Expression pattern analysis, Gene Ontology analysis, and pathway enrichment analysis were performed to investigate functional enrichment among up- or down-regulated genes using the DFCI Honey Bee Gene Index database, Cluster, AmiGO, and the KEGG database.

Because genes with similar expression patterns are functionally correlated, Cluster and Java Treeview software were used to perform cluster analysis of the gene expression patterns. DEGs among the samples were submitted to the Gene Ontology website for GO enrichment analysis and functional annotation. This analysis identified the GO terms that were significantly represented among the DEGs, and the genes were divided into three categories corresponding to the three domains covered by the ontology analysis: cellular component, molecular function, and biological process. The Bonferroni correction was used to adjust the p-values, and a p-value of 0.05 was used as the threshold. GO terms satisfying this condition were defined as significantly enriched GO terms among the DEGs. Because genes usually interact with each other in certain biological functions and to better understand the relationship and functions of the identified genes, the DEGs were submitted to the KEGG database for pathway enrichment analysis. This analysis identifies potentially affected pathways, such as the metabolic pathway or the signal transduction pathway, in the context of the whole genome background. Pathways with a Qvalue≤0.05 were considered significantly enriched, and the hyperlinks provided were used to access the detailed information in the KEGG database.

### 2.5 Validation of RNA-seq Data with qRT-PCR

To verify the data obtained by RNA-seq, qRT-PCR was performed in triplicate using SYBR Green (TaKaRa, Japan) and the ABI 7500 SDS system (Applied Biosystems, USA). Normalization against the initial total RNA concentration was performed using an Agilent 2100 Bioanalyzer for the reverse transcription step (Fermentas, Canada) and to synthesize the first cDNA strand for the control reactions. Gene-specific primers (GSPs, File S1) were designed using Primer-BLAST online and Oligo 7 software, ensuring that the primer pairs flanked an exon-exon boundary to allow differentiation of fragments amplified from genomic DNA. As one of the most stably expressed genes in various honey bee tissues and throughout different developmental stages, *β-actin* (406122) was selected as the endogenous reference gene to correct for sample variation in RT-PCR efficiency and errors in sample quantification [32].

PCR was first performed to verify the reliability of the GSPs when cDNA was used as the template, and the PCR conditions were optimized accordingly. A 10-fold dilution series of cDNA was used to construct a standard curve for the PCR efficiency (E: 0.8–1.2) and repeatability (R^2^≥0.98) (empirically). The qRT-PCR reactions (20 µl) were performed in 8-strip tubes (Axygen, USA)containing: 10 µl SYBR premix Ex Taq II (DRR082A, TaKaRa), 0.4 µl ROX II, 8 µM each primerand 1 µlcDNA. The qRT-PCR conditions were as follows: an initial step at 95°C (30 s), 40 cycles of 95°C (5 s) and 60°C (34 s) and an additional dissociation stage. A 9600 emulation run mode was used. No-template controls (NTCs) were performed along with each reaction. The data were collected at the 60°C (34 s) step.

The qRT-PCR data were expressed relative to the expression of *β-actin* using the 2^−ΔΔCt^ method, an independent-sample t-test available in SPSS software (Version 16.0, SPSS Inc.). A *p*-value of 0.01 was used to determine statistical significance [32].

## Results and Discussion

### 3.1 Morphological Development of HGs

Previous estimates of HG activity [33], [34] primarily relied on morphology and/or acinar size, with the implicit assumption that larger size corresponds to higher activity and the diameter reflects the amount of production thus giving an indication of gland activity [35], except in broodless bees, such as winter bees or bees in a swarm [36], [37], which typically have a low protein synthesis rate in the HGs [38], [39].

Previous studies indicated the existence of the honey bee brood that activates HGs protein synthesis in nurse bees, the secretions produced by the HGs depend on the needs of the hive [25]. However, the signal for worker bees turning into foragers is a deficit of work within the hive because the degeneration of the HGs [40], correspondingly HGs cell apoptosis [6], decreased rough endoplasmic reticulum and suppressed protein synthesis rates [41]. In these studies, an *in vitro* bioassay was used to measure protein synthesis in the glands of bees reared under treatment conditions; the colonies were divided into brood-right and broodless portions with single or double screens. The worker bees responded to the signal only if they had direct access to the brood. Regression analysis revealed a quadratic relationship between total protein content and the synthesizing activity of the HGs indicating that both undeveloped and fully developed HGs had less actively synthesizing protein than glands of intermediate sizes [42].

Ultrastructural changes in HGs cells have previously been described in the imaginal development of worker bees (*Apis mellifera* L.) [43], [44]. When the rates of protein synthesis were measured in nurse bees, low protein production was observed in foragers along with changes in the rough endoplasmic reticulum; in other words, protein production increased immediately after emergence, reached a maximum during the nursing phase (i.e. nurse bees), then decreased in field bees (i.e. foragers).

HGs reach maturation at 9 days, then subsequently shrink [45], [46]. Days 6 to 12 correspond to the peak interval of RJ secretion and the time during which the worker bees take care of the queen and her larvae [24]. In the present study, the workers were collected 3, 6, 9, 12, and 16 days after emergence by the experimental design. The diameters of the acini were used to assess morphology by one-way ANOVA in SPSS 16, and the values at 9 days were significantly greater than the values on other days (Figure S5, Table S3 in Document S1).

### 3.2 RNA-seq and Analysis of the Raw Data

To better understand the dynamic process of HG development, 30 HGs were pooled for each sample and homogenized for RNA isolation. A total of five Solexa cDNA libraries were constructed for deep sequencing analysis, and the statistics for each of the five samples are summarized in Table 1 (see Table S4 in Document S1 and Figure S6 for further details).

**Table 1:** Sample statistics.

Sample ID	1	2	3	4	5
**Age (Day)**	3	6	9	12	16
**Total RNA (µg)**	27.72	30.04	69.35	59.66	67.5
**RIN** *	8.1	9.4	8.3	7.7	7.6
**Clean Reads (%)**	3573941, 99.39%	3562730, 9.46%	3551541, 99.54%	3524453, 99.50%	3615558, 99.36%
**Unique Matched Genes** **	18378 (73.49%)	17785 (71.12%)	17065 (68.24%)	17105 (68.40%)	17995 (71.96%)
**Unigene_all** ***	20522 (100%)				

* Agilent Bioanalyzer 2100 RNA integrity number.

** The total unigene number in the DFCI Honey Bee Gene Index database is 25007; http://compbio.dfci.harvard.edu/cgi-bin/tgi/gimain.pl?gudb=honeybee.

*** The unique matched genes from all five samples cover the DFCI database completely.

### 3.3 Expression Level Analysis

**Figure 1:**
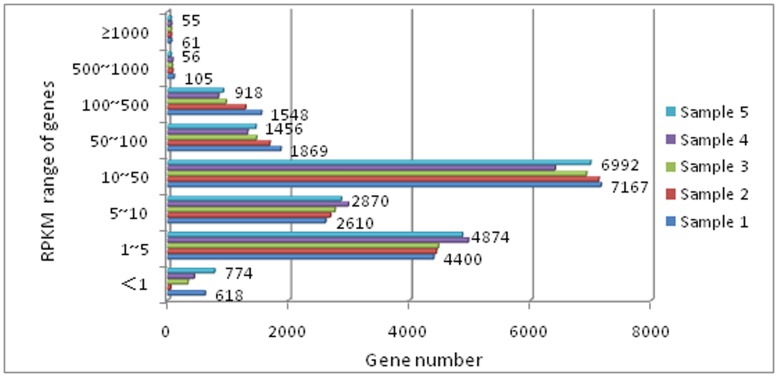
Abundance distribution of unigenes. The expression level ranges are as follows: [0, 10): low-expression genes; [10, 500): moderate-expression genes; and [500, ∞): high-expression genes. Large differences in abundance were observed for samples 1 and 5.

The gene expression levels were classified into eight grades according to their RPKM and set criteria to identify low-, moderate- and high-expression genes by experience (Figure 1 and Table S5 in Document S1). Considering the proportion of low-expression genes identified in samples 1 and 5 relative to the other samples, high-throughput Solexa sequencing permits the discovery of a wide range of transcriptomes and also provides a far more precise measurement of transcript levels [47].

Correlation analysis of technical replicates was used to evaluate the reliability and reproducibility of the experimental results, along with operational stability (Figure S7) as a strict formula of quality control.

### 3.4 Screening and Functional Analysis of DEGs

#### 3.4.1 DEGs common among the samples

**Figure 2:**
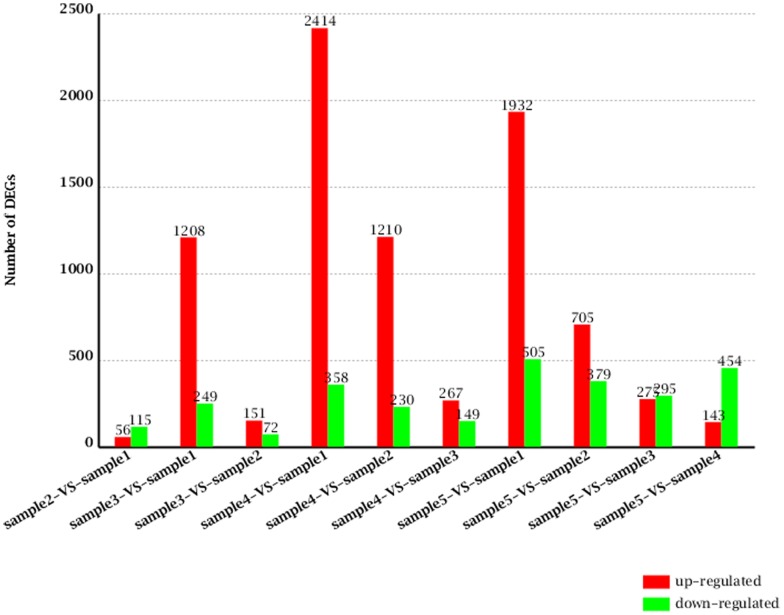
Up- and down-regulated DEGs in HGs. The x-axis indicates the pairwise comparisons of the five samples. The y-axis indicates the number of DEGs. Red represents up-regulated and green represents down-regulated.

**Figure 3:**
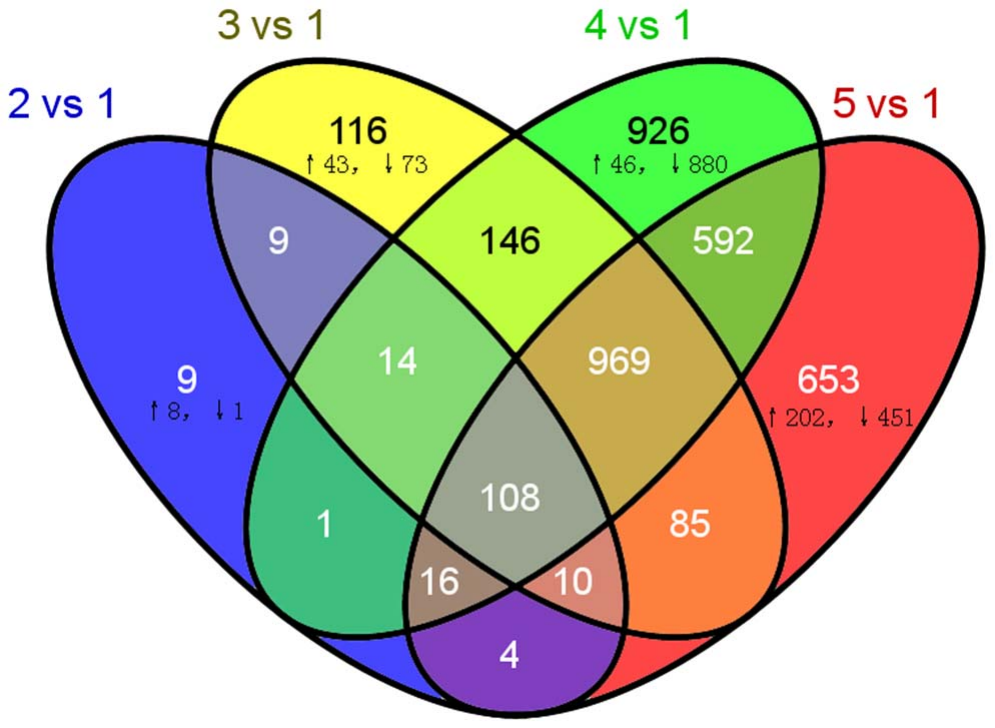
Venn analysis of the DEGs. The number of DEGs in the five samples named 2 vs. 1, 3vs. 1, 4vs. 1, and 5vs. 1. Numbers stands for genes expressed in each class. Up- and down- arrow represents the up and down regulated genes, respectively (FDR≤0.001).

Pairwise comparisons of the five samples were used to identify 3658DEGs (Figure 2). Sample 1 was used as the baseline for reducing the background noise corresponding to genes unrelated to HGs secretion, and the other samples were compared to sample 1 to identify the filtered DEGs. The Venn diagram in Figure 3 shows the specifically expressed genes and the 108 shared DEGs (2.95%) (Figure S8); the 44 annotated DEGs (41%) are also noted.

**Figure 4:**
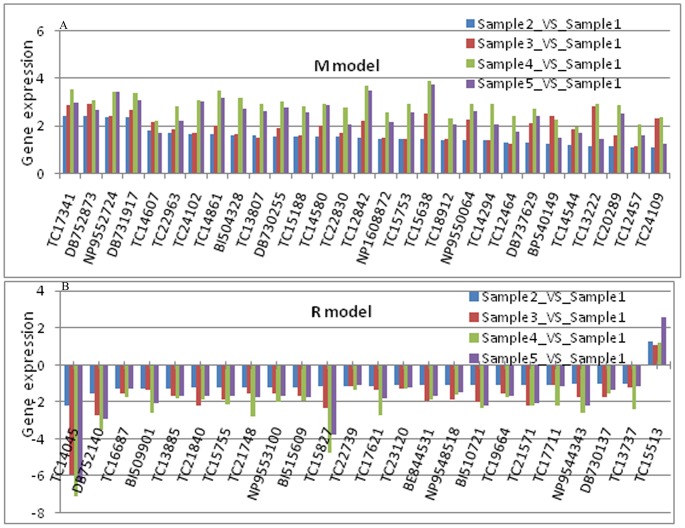
DEGs of the match model (M) and reverse model (R) in samples. Genes with their expression profiles showed in panel A (M) and B (R).

According to the established HGs development and secretion model (an initial increase followed by a decrease, with a peak at day 9), two groups of DEGs (matched, M, and reversed, R) were identified (Figure 4A, 4B and Table S6 in Document S1). There were 29 and 24 genes in the M and R groups, respectively.

Expression profile analysis of genes related to the cytoskeleton, enzyme activity, organelles, membranes, signaling and ion channels was performed using enrichment analysis for titin, troponin T, myosin, Muscle-specific protein 20, regucalcin and Sodium/potassium-transporting ATPase subunit alpha. Most of these genes were up-regulated by more than 5-fold on days 9 and 12, indicating the relationship between gene function and cell morphology. A total of 9 DEGs (DB752873, TC22830, DB737629, DB731917, TC24102, NP1608872, TC18912, BE844531, and TC14544) were selected for further discussion.

Regucalcin (TC14544) has been shown to play a multifunctional role in many cell types as a regulatory protein. It plays a critical role in the intracellular signaling system that maintains intracellular Ca^2+^ homeostasis in the plasma membrane, endoplasmic reticulum, and mitochondria of many cells. This regulation has a reversible effect on Ca^2+^-induced activation, the inhibition of many enzymes [48], and the musculation of acini.

Titin and troponin are both regulated by the calcium concentration in cells. Titin (DB752873, TC22830, DB737629) is a very large protein encoded by the TTN gene [49] and is important for the contraction of striated muscle tissues. Titin is also involved in various physiological processes that underlie the passive elasticity of muscle. The expression profile of titin was found to be consistent among the different samples and to correspond with HG development and activity. Protein transport and secretion are dependent on the musculation of acini; accordingly, an up-regulation of gene expression was observed in the yield stage and was followed by a decrease in gene expression. Troponin (DB731917, TC24102, NP1608872, TC18912) is a complex of three regulatory proteins (troponin C, troponin I, and troponin T) that are important for muscle contraction, which is dependent on the intracellular calcium concentration.

Sodium/potassium-transporting ATPase subunit beta-2-like (BE844531) is an integral membrane protein that is responsible for establishing and maintaining the electrochemical gradients of Na^+^ and K^+^ ions across the plasma membrane. These gradients are essential for osmoregulation, sodium-coupled transport of a variety of organic and inorganic molecules, and the electrical excitability of nerves and muscles. ATP hydrolysis drives the transport process and provides energy for cells.

Several methods for the identification of DEGs in honey bee have been reported. These methods include sequencing of cDNA libraries, suppression subtractive hybridization, Solexa/Illumina digital gene expression tag profiling [50] and high-throughput RNA-seq [51]. Proteomic analysis of HGs development previously identified differentially expressed proteins on different days of emergence and demonstrated that major royal jelly proteins (MRJPs/Yellow) and other key node proteins were compelling candidates for parsing the relationship between proteins and eusocial behavior in development [24].

The MRJPs gene family encodes a group of closely related proteins that share a common evolutionary origin with the duplication of Yellow protein [52] of *Drosophila melanogaster* and constitute approximately 90% of all RJ proteins [24], [53]–[56]. The MRJPs are the most abundant form of the identified proteins, increasing from 8.7% to 46.8% from day 1 to day 20 [24], and appear to have evolved a novel nutritional function in the honey bee. Royalactin is derived from the monomeric glycoprotein (57-kDa) MRJP1 [57], which has been identified as the key protein that triggers caste differentiation through an EGFR-mediated signaling pathway and produces female dimorphism in the colony (in the bee and fruit fly) [58]. The orthologous MRJP protein subfamily and Yellow protein family may have coincided with the evolution of honey bee eusociality [59]. To our knowledge, there are no secretory proteins in the HGs of workers at the emerging stage, which is consistent with an endogenous role of MRJPs in HGs.

#### 3.4.2 Specific DEGs in samples

In the present study, many DEGs with distinct expression patterns in each of the samples were identified (Figure 4). These include Ribosome biogenesis protein BOP1 (TC17720) and β-dehydrogenase (TC14601), which were expressed on day 6; *MRJP1*, *3*, and *4* (TC17816, TC22893, TC12751) and *yellow-e2* and *3* (BI514641, TC14541, DB739842), which were expressed only on day 9 in a manner consistent with the proteomic analysis of HGs [24] validating that MRJPs should be the main contents of the RJ. Interestingly, major royal jelly protein 2 precursor gene (DB779314), a member of *MRJPs* family, was identified on day 3 with a tiny expression pattern, suggesting that the honeybees in early stage are likely to synthesize MRJPs, which played a certain role in biological function, such as nursing the brood and trigging the caste differentiation [51]. Glucose oxidase (BP539841) [60], [61], phospholipase (BP539841), exonuclease (TC15395), glucocerebrosidase (TC19526), carboxypeptidase M (BI506661) and RasGTPase activator (BI507785, NP9553760, TC17753) were expressed on day 12. In consideration of the slight role change as they age, worker bees deposit the nectar and pollen from returning field bees into cells, and maintain the colony with the ripened honey and the pollen [62]. Glucose dehydrogenase (NP9552746, TC21807), glucohydrolase (NP9553382), α-amylase (DB772014), α-glucosidase (TC19959) and *yellow-h* (TC12331) were expressed on day 16. Previous reports have demonstrated that α-glucosidase is the critical enzyme which is synthesized by HGs and converts nectar into honey for the forager [22], in parallel with glucose dehydrogenase [26], glucohydrolase, and α-amylaserelevant to carbohydrate-metabolizing enzymes that may have particular roles in the honey bee [63]. In this study, α-glucosidase was detected on day 16, suggesting the upcoming task switch from nursing to foraging. DEGs related to metabolic enzymes and energy production provided substrates and complements to ensure the formation of HGs and other biological phenomenon [64].

The present findings about DEGs in the HGs expand our understanding of gene function and provide candidate genes for genetic manipulation and further study. The varied distribution of down-regulated DEGs, such as α-amylase suggested that HGs shrinking corresponds to task switching. Partial up-regulation of DEGs such as glucose dehydrogenase (NP9552746) and glucohydrolase (NP9553382) coincided with task shifting from nurse bees to forager bees. Nurse bees generally remain in the hive to feed and care for the queen and her larvae, while foragers are responsible for gathering pollen and nectar. It was previously demonstrated that reversible DNA methylation patterns in brain cells yield behavioral changes [12]. The present findings of up- or down-regulation of genes such as methyltransferase (TC21859), protein arginine N-methyltransferase 5 (NP9553857), methylosome subunit pICln (TC13507) and aminomethyltransferase (NP9553401) specifically on day 16 are consistent with the findings of previous studies.

### 3.5 Expression Pattern Analysis of DEGs

**Figure 5:**
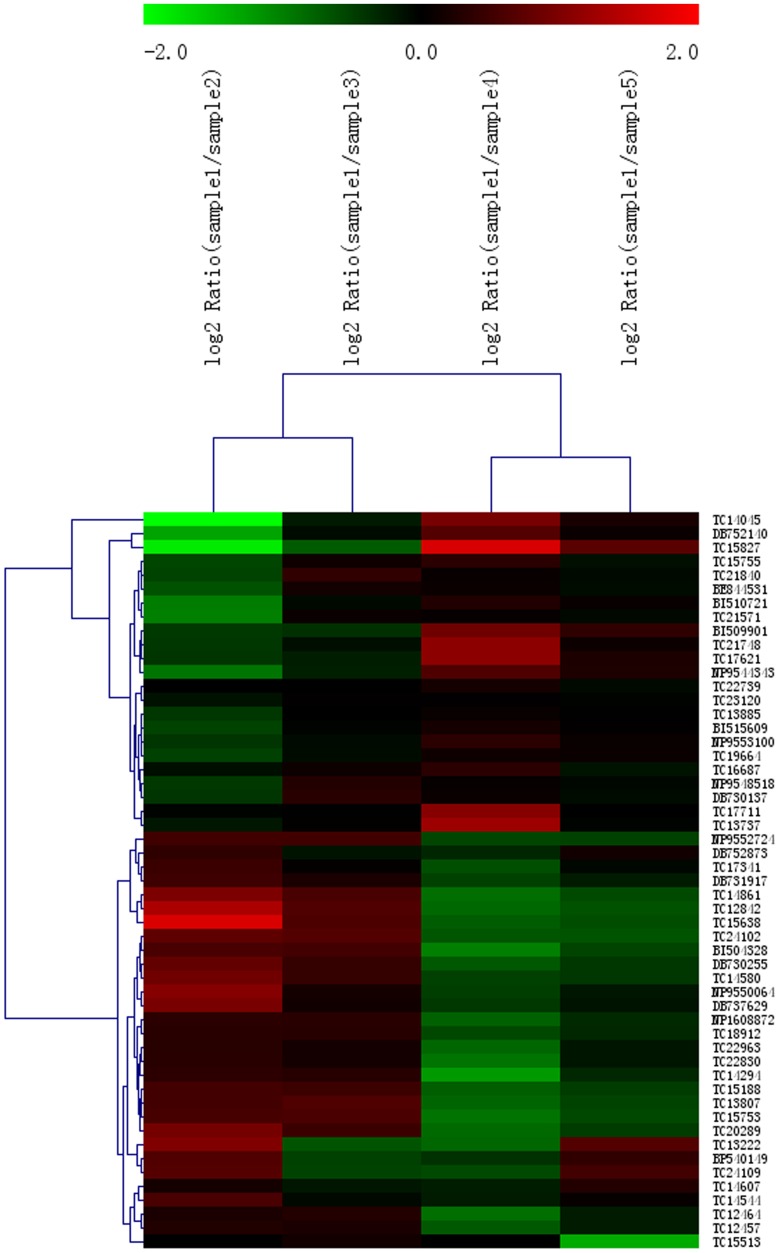
Cluster analysis of DEG levels. Each column represents an experimental condition relative to sample 1, and each row represents a gene. Expression differences are shown in different colors. Red and green indicate up-regulation and down-regulation, respectively.

The DEGs identified in the present study were divided into two groups. Less than half of the DEGs had high expression on days 3 and 6 and low expression on day 12, a pattern consistent with HG secretion (Figure 5). The other DEGs (more than half of all DEGs) were consistent with the characteristics of HGs development: high expression on days 3 and 6, in accordance with the comprehensive morphology and preparations for synthesizing and producing action, and low expression on days 12 and 16, which is consistent with the decreased activity of acinar cells.

### 3.6 Gene Ontology Analysis of DEGs

By providing information about cellular and subcellular localization, gene functions, and biological processes, the GO and KEGG databases highlight the complexity of living organisms and their surroundings [65].

**Figure 6:**
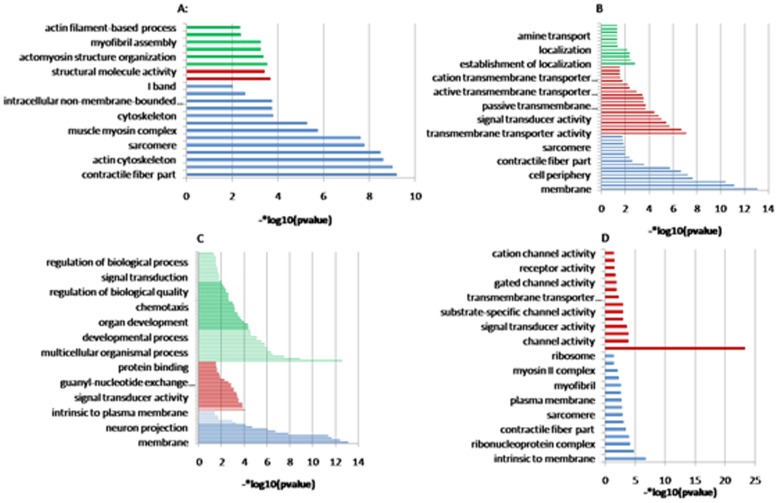
GO enrichment analysis. The GO terms were classified into 3 categories from the GO database and are ranked by p-value. Green indicates biological processes, red indicates molecular functions, and blue indicates cellular components. (A) Sample 2 vs. 1, (B) Sample 3 vs. 1, (C) Sample 4 vs. 1, (D) Sample 5 vs. 1.

GO analysis was performed to identify the functional components that are regulated in a manner consistent with HGs development. GO enrichment (Figure 6A to 6D and Table S7 in Document S1) indicated that genes related to structure, the cytoskeleton, cellular morphogenesis, membranes, and signaling were enriched, specifically those under the GO terms “structural molecule activity”, “plasma membrane”, and “system development”.

### 3.7 Pathway Enrichment Analysis of DEGs

Approximately 110 pathways were identified, containing more than 1000 DEGs. Table S8 in Document S1 lists the most abundant differentially expressed signaling pathways shared among the samples and the ranking of the top five pathways. The differentially regulated transcripts implicated in the general mechanisms of energy metabolism, catalytic activity, amino acid metabolism, protein synthesis and transport are shown. We were particularly interested in transcripts involved in the processes such as the ribosome pathway and protein processing in the endoplasmic reticulum. The consumption of protein-rich pollen and carbohydrates could satisfy the energy and material requirements of HGs development and the secretion of larval food [66]. Some key signaling pathways may warrant attention for their roles in disease and may represent potential targets for tumor therapy.

#### 3.7.1 Ribosomes are the cellular factories responsible for producing proteins and exhibiting dynamic variation consistent with HGs development

Most tissue development (e.g., HGs, ovaries, and fat bodies) occurs within the first week after emergence [67] and ribosomes pathway is perhaps the most commonly activated signaling pathway, proteins constituted the main active ingredients of RJ. Because these proteins perform a vast array of functions throughout the life cycle, compromised ribosomes may result in disease or even death [68]. In the present study, more than 70 DEGs implicated in ribosome biogenesis were enriched in the ribosome pathway (ame03010); thus, the regulation of ribosome biogenesis appears to be consistent with the physiological development and activities of HGs. The ribosome pathway DEGs are listed in File S2. Most of the DEGs in ribosome pathway upregulated on day 9 compared to day 16 (i.e. Sample 5 vs. 3), suggesting that HGs might be able to increase protein synthesis for RJ secretion. Coincidentally, the expression patterns of those DEGs were well matched with the yield trait of worker bees of certain age. Furthermore, a sufficient protein synthesis and reservation might have the benefit under busy work and various external environments.

#### 3.7.2 The PI3K-Aktsignalingpathway plays a central role in regulating diverse downstream pathways associated with cellular processes such as mitosis, apoptosis, proliferation, cell cycling, protein synthesis and glucose metabolism [69]

Activated Akt modulates the function of numerous substrates involved in the regulation of cell survival, cell cycle progression and cellular growth [70]. PI3K activation induces AKT, which in turn activates TOR.

#### 3.7.3 The TOR pathway involves a nutrient- and energy-sensing kinase that controls organismal growth [71], metabolism [18], reproduction, and lifespan [72], [73]

Central to the pathway is the TOR protein, a member of the phosphoinositide 3-kinase (PI3K)-related protein kinase (PIKK) family [74], [75]this pathway has also been implicated in the regulation of the division of labor between worker bees at the social level [17], [76], [77]. A recent study found that the inhibition of insulin receptor substrate expression in peripheral tissues (as opposed to central brain tissues) results in the collection of pollen rather than nectar by foragers [78]. The insulin-like growth factor-binding protein (NP9547607, TC14287, TC21952) was highly expressed in the sample corresponding to day 16. Interestingly, all of the DEGs included in the TOR signaling pathway were downregulated along with the HGs development (File S3), indicating that depression of the morphology, activities, and physiological development of HGs [18], presumably were downregulated through the biological process of downstream cascades and physiological development.

Corresponding to the global RNA-seq profiling, partial expression pattern of DEGs did not always match well to the biological rhythm of HGs we assumed.

### 3.8 Relative qRT-PCR Analysis

Five randomly selected DEGs and thirteen DEGs related to protein metabolism, enzymes, or metabolites were validated by qRT-PCR. The reactions were performed using the ABI 7500 SDS system with SYBR Green. The qRT-PCR results were basically consistent with the RNA-seq data (Table 2). *Actin* a housekeeping gene, was used for internal control gene as described in previous studies [79], [80].

**Table 2:** Validation of selected genes by qRT-PCR.

Gene ID	RNA-seq (Ratio-RPKM)					qRT-PCR (fold-change)				
	Samp 1/1	Samp 2/1	Samp 3/1	Samp 4/1	Samp 5/1	Samp 1/1	Samp 2/1	Samp 3/1	Samp 4/1	Samp 5/1
**TC14045**	1	0.214043	0.016566	0.007441	0.012394361	1	0.24	0	0	0
**TC16687**	1	0.407727	0.34737	0.297202	0.41803829	1	0.78	0.5	0.48	3.91
**NP9548518**	1	0.462605	0.27878	0.326666	0.35468766	1	0.72	0.45	0.57	5.05
**NP9552724**	1	5.23677	5.25408	10.90262	10.67966242	1	5.86	6.44	16.15	35.77
**TC12464**	1	2.474746	2.372389	5.271425	3.329252313	1	4.17	3.71	6.32	27.5
**DB731917**	1	5.217156	6.327097	10.5259	8.571618454	1	2.385	1.956	7.527	4.563
**DB752873**	1	5.366218	7.733118	8.589137	6.265073852	1	4.377	4.857	7.548	9.533
**TC12842**	1	2.818196	4.628154	12.68371	11.20154684	1	3.638	6.489	30.889	130.147
**TC13222**	1	2.17906	6.990682	7.691906	2.780256289	1	2.505	6.878	10.225	17.765
**TC14861**	1	3.095895	4.088278	11.13479	9.149453433	1	3.297	4.098	17.125	23.474
**TC17341**	1	5.430102	7.230967	11.54842	7.747852525	1	2.239	0.977	4.07	5.042
**TC20289**	1	2.178593	2.992302	7.368132	5.730396673	1	2.092	3.251	20.952	51.162
**TC22830**	1	2.894723	3.238871	6.856874	4.107697675	1	2.352	3.015	13.804	22.753
**TC22963**	1	3.280976	3.637316	7.166767	4.594116452	1	3.701	5.4	37.792	55.87
**DB752140**	1	0.341965	0.148873	0.089161	0.132012092	1	0.437	0.085	0.106	0.155
**TC15827**	1	0.439908	0.199672	0.03737	0.074696191	1	0.448	0.097	0.025	0.215
**TC17621**	1	0.451108	0.394673	0.155119	0.287087699	1	0.587	0.344	0.155	1.423
**TC21748**	1	0.428985	0.343331	0.146873	0.293573402	1	0.493	0.383	0.181	1.382

Gene IDs refer to the corresponding cDNAs from Genbank. qRT-PCR was performed on each sample in triplicate. *β-actin* was used an endogenous control. Ratio-RPKM used for comparing the difference of gene expression among samples and validation by qRT-PCR to show a certain consistent between the two methods except several inconsistencies. It may be attributed to the experimental errors which caused by the biological replicas deficiency. Only one pooled RNA sequenced for each sample due to the limitations of specimen collection that might cause the experimental errors unpredictably. We will sequence more samples to eliminate the errors in the further research.

In summary, transcriptomic analysis was used to investigate the regulation of HGs in nurse bees at different ages. The data revealed that up- and down-regulated genes were involved in metabolism, protein synthesis, and the transport signaling pathway, thereby providing insight into the molecular mechanisms of the development and secretion of RJ. The results are of importance for further study to improve honey bee breeding techniques and help ensure stable yields of RJ with high quality traits.

## Supporting Information

Figure S1 Classification of Raw Reads. Sample 1 to 5 stand for Day 3 to 16, respectively; Bright green: raw reads containing adapters; Red: raw reads containing unknown bases; Dark green: low quality reads; Blue: clean reads used in the next steps.
(DOCX)

Figure S2 The sequencing saturation of samples. X-axis is number of clean reads, y-axis is the percentage of identified genes. When number of clean reads reaches about 3 M or higher, the number of detected genes almost ceases to increase.
(DOCX)

Figure S3 Distribution of reads on reference genes of samples. X-axis is relative position in gene, Y-axis is number of reads. RNA fragmentation (black curve) provides more even coverage along the gene body, but is relatively depleted for both the 5′ and 3′ ends. Reads should be evenly distributed on reference genes, otherwise it means the randomness is not good and this will affect following analysis.
(DOCX)

Figure S4 Genes, coverage analysis of samples. Gene coverage is calculated as the percentage of a gene covered by reads. This value is equal to the ratio of the base number in a gene covered by unique mapping reads to the total base number of coding region in that gene.
(DOCX)

Figure S5 Development pattern of HGs acini. Panel A represents ESEM profiles of HGs on day 3, 6, 9, 12, and 16 at 100–400× magnification, respectively. The numbers indicates the HGs after the eclosion. Panel B is the HGs acini mean diameter. Asterisks indicate the statistically significant differences between the mean diameter of acini at each development stage (n≥34, *p*<0.05). Note: The newly added figures were magnified in equal proportion (400×) based on Figure 1. The full-scale original drawing of the panel A was also supplied as Additional File 4.
(DOCX)

Figure S6 Venn chart of all unique matched genes covered to the DFCI Amel database.
(DOCX)

Figure S7 Experimental repeatability analysis. Experimental repeatability is defined by the correlation of technical replicates. The closer the value of correlation gets to 1, the better the repeatability between two parallel experiments.
(DOCX)

Figure S8 Genes shared expressed in the 5 samples (108 in all). Genes with their expression profiles showed in this figure similar to Figure 5, X-axis is gene ID, Y-axis is the expression (Log_2_ Ratio); 2\1, 3\1, 4\1, 5\1 stand for sample 2 vs 1, sample 3 vs 1, sample 4 vs 1, sample 5 vs 1, respectively.
(DOCX)

File S1 Gene special primers for qPCR.
(XLSX)

File S2 Ribosome pathway DEGs.
(XLSX)

File S3 TOR signaling pathway DEGs.
(XLSX)

Document S1 Table S1–S8. Table S1, Components of fresh royal jelly (GB9697-2008). Table S2, The vitamin content (in mg) of one gram according to the United States Department of Agriculture. Table S3, Development pattern of Hypopharyngeal gland. Table S4, Sample demographics. Table S5, RPKM Range of unigenes. Table S6, DEGs of M and R model. Table S7, Top 20 of GO Enrichment analysis in samples (p≤0.05). Table S8, The most abundant differentially expressed signalling pathways shared in the samples except sample 1 (Top 5 in bold).
(DOCX)
