# Supplementary material for: Transcriptome Sequencing Analysis Reveals the Regulation of the Hypopharyngeal Glands in the Honey Bee, Apis mellifera carnica Pollmann
Source: PLoS One. 2013 Dec 10;8(12):e81001. doi: 10.1371/journal.pone.0081001 (PMC3858228; doi:10.1371/journal.pone.0081001)
Supplement: Figure S1 — Classification of Raw Reads. Sample 1 to 5 stand for Day 3 to 16, respectively; Bright green: raw reads containing adapters; Red: raw reads containing unknown bases; Dark green: low quality reads; Blue: clean reads used in the next steps. (DOCX) [file pone.0081001.s001.docx]

**Figure S1 Classification of Raw Reads.** Sample 1 to 5 stand for Day 3 to 16, respectively; Bright green: raw reads containing adapters; Red: raw reads containing unknown bases; Dark green: low quality reads; Blue: clean reads used in the next steps.
