# Supplementary material for: Transcriptome Sequencing Analysis Reveals the Regulation of the Hypopharyngeal Glands in the Honey Bee, Apis mellifera carnica Pollmann
Source: PLoS One. 2013 Dec 10;8(12):e81001. doi: 10.1371/journal.pone.0081001 (PMC3858228; doi:10.1371/journal.pone.0081001)
Supplement: Figure S2 — The sequencing saturation of samples. X-axis is number of clean reads, y-axis is the percentage of identified genes. When number of clean reads reaches about 3 M or higher, the number of detected genes almost ceases to increase. (DOCX) [file pone.0081001.s002.docx]

**Figure S2 The sequencing saturation of samples.** X-axis is number of clean reads, y-axis is the percentage of identified genes. When number of clean reads reaches about 3M or higher, the number of detected genes almost ceases to increase.
