# Supplementary material for: Transcriptome Sequencing Analysis Reveals the Regulation of the Hypopharyngeal Glands in the Honey Bee, Apis mellifera carnica Pollmann
Source: PLoS One. 2013 Dec 10;8(12):e81001. doi: 10.1371/journal.pone.0081001 (PMC3858228; doi:10.1371/journal.pone.0081001)
Supplement: Figure S3 — Distribution of reads on reference genes of samples. X-axis is relative position in gene, Y-axis is number of reads. RNA fragmentation (black curve) provides more even coverage along the gene body, but is relatively depleted for both the 5′ and 3′ ends. Reads should be evenly distributed on reference genes, otherwise it means the randomness is not good and this will affect following analysis. (DOCX) [file pone.0081001.s003.docx]

**Figure S3 Distribution of reads on reference genes of samples.** X-axis is relative position in gene, Y-axis is number of reads. RNA fragmentation (black curve) provides more even coverage along the gene body, but is relatively depleted for both the 5' and 3' ends. Reads should be evenly distributed on reference genes, otherwise it means the randomness is not good and this will affect following analysis.
