# Supplementary material for: Transcriptome Sequencing Analysis Reveals the Regulation of the Hypopharyngeal Glands in the Honey Bee, Apis mellifera carnica Pollmann
Source: PLoS One. 2013 Dec 10;8(12):e81001. doi: 10.1371/journal.pone.0081001 (PMC3858228; doi:10.1371/journal.pone.0081001)
Supplement: Figure S4 — Genes, coverage analysis of samples. Gene coverage is calculated as the percentage of a gene covered by reads. This value is equal to the ratio of the base number in a gene covered by unique mapping reads to the total base number of coding region in that gene. (DOCX) [file pone.0081001.s004.docx]

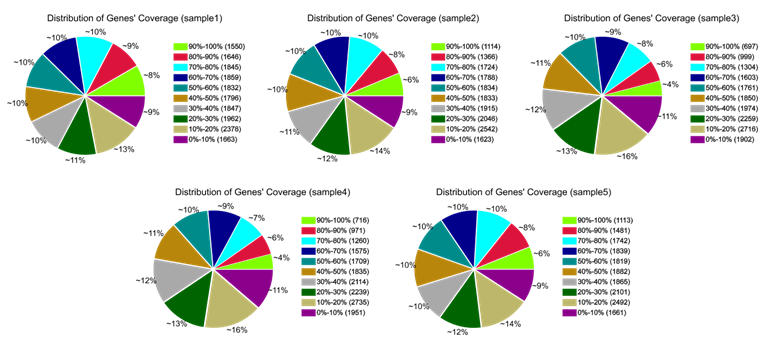


**Figure S4 Genes^,^ coverage analysis of samples.** Gene coverage is calculated as the percentage of a gene covered by reads. This value is equal to the ratio of the base number in a gene covered by unique mapping reads to the total base number of coding region in that gene.
