# Supplementary material for: Transcriptome Sequencing Analysis Reveals the Regulation of the Hypopharyngeal Glands in the Honey Bee, Apis mellifera carnica Pollmann
Source: PLoS One. 2013 Dec 10;8(12):e81001. doi: 10.1371/journal.pone.0081001 (PMC3858228; doi:10.1371/journal.pone.0081001)
Supplement: Figure S5 — Development pattern of HGs acini. Panel A represents ESEM profiles of HGs on day 3, 6, 9, 12, and 16 at 100–400× magnification, respectively. The numbers indicates the HGs after the eclosion. Panel B is the HGs acini mean diameter. Asterisks indicate the statistically significant differences between the mean diameter of acini at each development stage (n≥34, p<0.05). Note: The newly added figures were magnified in equal proportion (400×) based on Figure 1. The full-scale original drawing of the panel A was also supplied as Additional File 4. (DOCX) [file pone.0081001.s005.docx]

**Figure 1. Development pattern of HGs acini.** Panel A represents ESEM profiles of HGs on day 3, 6, 9, 12, and 16 at 100-400× magnification, respectively. The numbers indicates the HGs after the eclosion. Panel B is the HGs acini mean diameter. Asterisks indicate the statistically significant differences between the mean diameter of acini at each development stage (n≥34, *p* < 0.05).Note: The newly added figures were magnified in equal proportion (400×) based on Figure 1. The full-scale original drawing of the panel A was also supplied as Additional File 4.
