# Supplementary figures and images for: Transcriptome Sequencing Analysis Reveals the Regulation of the Hypopharyngeal Glands in the Honey Bee, Apis mellifera carnica Pollmann
Source: PLoS One. 2013 Dec 10;8(12):e81001. doi: 10.1371/journal.pone.0081001 (PMC3858228; doi:10.1371/journal.pone.0081001)

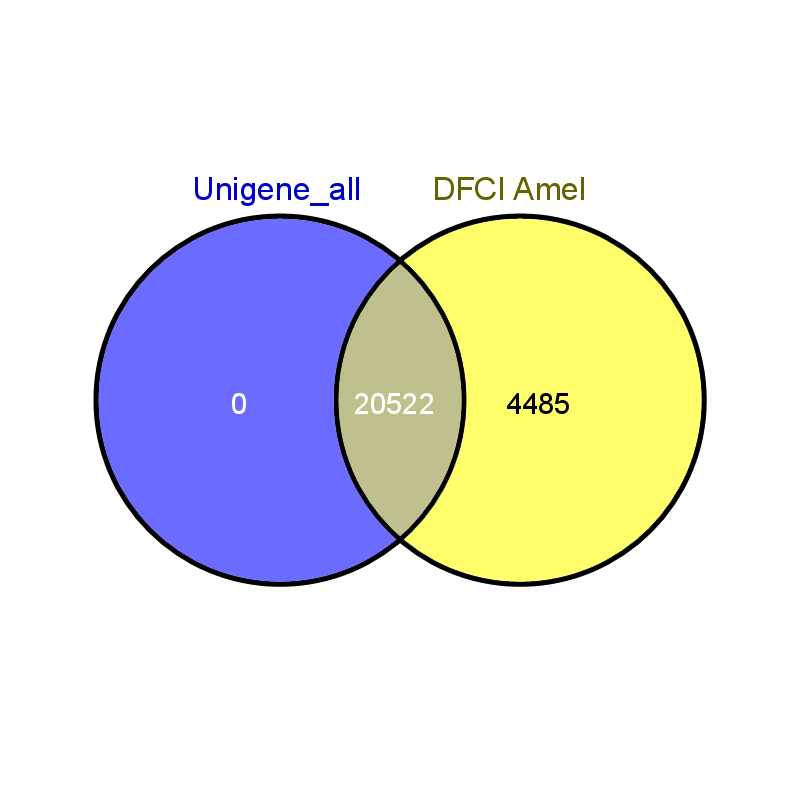


**Figure S6** **Venn chart of all unique matched genes covered to the DFCI Amel database.**

Supplement: Figure S6 — Venn chart of all unique matched genes covered to the DFCI Amel database. (DOCX) [file pone.0081001.s006.docx]
