# Supplementary material for: Transcriptome Sequencing Analysis Reveals the Regulation of the Hypopharyngeal Glands in the Honey Bee, Apis mellifera carnica Pollmann
Source: PLoS One. 2013 Dec 10;8(12):e81001. doi: 10.1371/journal.pone.0081001 (PMC3858228; doi:10.1371/journal.pone.0081001)
Supplement: Figure S7 — Experimental repeatability analysis. Experimental repeatability is defined by the correlation of technical replicates. The closer the value of correlation gets to 1, the better the repeatability between two parallel experiments. (DOCX) [file pone.0081001.s007.docx]

**Figure S7 Experimental repeatability analysis.** Experimental repeatability is defined by the correlation of technical replicates. The closer the value of correlation gets to 1, the better the repeatability between two parallel experiments.
