# Supplementary material for: Transcriptome Sequencing Analysis Reveals the Regulation of the Hypopharyngeal Glands in the Honey Bee, Apis mellifera carnica Pollmann
Source: PLoS One. 2013 Dec 10;8(12):e81001. doi: 10.1371/journal.pone.0081001 (PMC3858228; doi:10.1371/journal.pone.0081001)
Supplement: Figure S8 — Genes shared expressed in the 5 samples (108 in all). Genes with their expression profiles showed in this figure similar to Figure 5, X-axis is gene ID, Y-axis is the expression (Log2 Ratio); 2\1, 3\1, 4\1, 5\1 stand for sample 2 vs 1, sample 3 vs 1, sample 4 vs 1, sample 5 vs 1, respectively. (DOCX) [file pone.0081001.s008.docx]

**Figure S8** **Genes shared expressed in the 5 samples (108 in all).** Genes with their expression profiles showed in this figure similar to Figure 5, X-axis is gene ID, Y-axis is the expression (Log_2_ Ratio); 2\1, 3\1, 4\1, 5\1 stand for sample 2 vs 1, sample 3 vs 1, sample 4 vs 1, sample 5 vs 1, respectively.
