# Supplementary material for: Transcriptome Sequencing Analysis Reveals the Regulation of the Hypopharyngeal Glands in the Honey Bee, Apis mellifera carnica Pollmann
Source: PLoS One. 2013 Dec 10;8(12):e81001. doi: 10.1371/journal.pone.0081001 (PMC3858228; doi:10.1371/journal.pone.0081001)
Supplement: Document S1 — Table S1–S8. Table S1, Components of fresh royal jelly (GB9697-2008). Table S2, The vitamin content (in mg) of one gram according to the United States Department of Agriculture. Table S3, Development pattern of Hypopharyngeal gland. Table S4, Sample demographics. Table S5, RPKM Range of unigenes. Table S6, DEGs of M and R model. Table S7, Top 20 of GO Enrichment analysis in samples (p≤0.05). Table S8, The most abundant differentially expressed signalling pathways shared in the samples except sample 1 (Top 5 in bold). (DOCX) [file pone.0081001.s012.docx]

**Table S1** Components of fresh royal jelly (GB9697-2008)

| Index | Water (≤) | 10-HAD (≥) | Protein | Total sugar  (glucose) | Ash | Acidity  (1 mol/L, mL/100 g) | Starch |
| --- | --- | --- | --- | --- | --- | --- | --- |
| Excellent | 67.5 | 1.8 | 11-16 | 15 | 1.5 | 30-53 | Negative |
| Qualified | 69.0 | 1.4 |  |  |  |  |  |

**Table S2** The vitamin content (in mg) of one gram according to the United States Department of Agriculture

| VB1 (Thiamin ) | VB2 (Riboflavin) | VB6 (Pyridoxine) | Niacin (Nicotinic acid) | Pantothenic Acid |
| --- | --- | --- | --- | --- |
| 1.5-7.4 | 5.3-10.0 | 2.2-10.2 | 91.0-149.0 | 65.0 -200.0 |
| Biotin | **Inositol** | **Folic Acid** | **Vitamin C** | **Vitamin E** |
| 0.9 -3.7 | 78.0-150.0 | 0.16-0.50 | Trace amounts | None |

**Table S3** Development pattern of Hypopharyngeal gland

| Day old | Daimeter(μm，Mean±SD) | Sig |
| --- | --- | --- |
| 6 | 66.20±6.65 | A |
| 9 | 87.19±15.06 | B** |
| 12 | 70.29±8.69 | A |
| 16 | 71.16±9.16 | A |
| **P<0.01,n≥34 | | |

**Table S4** Sample demographics

| Sample ID | 1 | 2 | 3 | 4 | 5 |
| --- | --- | --- | --- | --- | --- |
| Age (Day) | 3 | 6 | 9 | 12 | 16 |
| Total RNA (μg) | 27.72 | 30.04 | 69.35 | 59.66 | 67.5 |
| RIN* | 8.1 | 9.4 | 8.3 | 7.7 | 7.6 |
| Tatal reads | 3573941 | 3562730 | 3551541 | 3524453 | 3615558 |
| Total mapped reads | 2601641(72.79%) | 2827291(79.36%) | 3040631(85.61%) | 3015495(85.56%) | 2932892(81.12%) |
| Clean Reads (%) | 3573941(99.39%) | 3562730(99.46%) | 3551541(99.54%) | 3524453(99.50%) | 3615558(99.36%) |
| perfect match | 2249624(62.95%) | 2465520(69.20%) | 2685084(75.60%) | 2651083(75.22%) | 2543413(70.35%) |
| <=2 mismatch | 352017(9.85%) | 361771(10.15%) | 355547(10.01%) | 364412(10.34%) | 389479(10.77%) |
| Unique match | 1697792(47.50%) | 1518649(42.63%) | 1207515(34.00%) | 1344138(38.14%) | 1815654(50.22%) |
| Multi-position match | 903849(25.29%) | 1308642(36.73%) | 1833116(51.61%) | 1671357(47.42%) | 1117238 (30.90%) |
| Total unmapped reads | 972300(27.21%) | 735439(20.64%) | 510910(14.39%) | 508958(14.44%) | 682666(18.88%) |
| Total Gene Number | 25007** | | | | |
| Unique Matched Genes | 18378(73.49%) | 17785(71.12%) | 17065(68.24%) | 17105(68.40%) | 17995(71.96%) |
| Unigene_all*** | 25022 (100%) | | | | |

*Agilent BioAnalyzer 2100 RNA integrity number after. **DFCI database, <http://compbio.dfci.harvard.edu/cgi-bin/tgi/gimain.pl?gudb=honeybee>

***Unique matched genes of all the five samples are covered to the DFCI Amel database completely.

**Table S5** RPKM Range of unigenes

| RPKM range of unigenes | | Gene number | | | | | Rate | | | | | Ave. |
| --- | --- | --- | --- | --- | --- | --- | --- | --- | --- | --- | --- | --- |
|  |  | Sample 1 | Sample 2 | Sample 3 | Sample 4 | Sample 5 | Sample 1 | Sample 2 | Sample 3 | Sample 4 | Sample 5 |  |
| Low expression | ＜1 | 618 | 48 | 333 | 437 | 774 | 0.4151 | 0.4121 | 0.4438 | 0.4908 | 0.4734 | 0.4470 |
|  | 1~5 | 4400 | 4442 | 4476 | 4972 | 4874 |  |  |  |  |  |  |
|  | 5~10 | 2610 | 2691 | 2765 | 2986 | 2870 |  |  |  |  |  |  |
| Moderate expression | 10~50 | 7167 | 7129 | 6925 | 6409 | 6992 | 0.5759 | 0.5798 | 0.5483 | 0.5009 | 0.5205 | 0.5451 |
|  | 50~100 | 1869 | 1687 | 1467 | 1320 | 1456 |  |  |  |  |  |  |
|  | 100~500 | 1548 | 1287 | 964 | 839 | 918 |  |  |  |  |  |  |
| High expression | 500~1000 | 105 | 83 | 75 | 81 | 56 | 0.009 | 0.0081 | 0.0079 | 0.0083 | 0.0062 | 0.0079 |
|  | ≥1000 | 61 | 58 | 60 | 61 | 55 |  |  |  |  |  |  |

**Table S6** DEGs of M and R model

| GeneID | Gene name | Accession number | Descriptiton | Sample 2 VS 1 | | Sample 3 VS 1 | | Sample 4 VS 1 | | Sample 5 VS 1 | |
| --- | --- | --- | --- | --- | --- | --- | --- | --- | --- | --- | --- |
|  |  |  |  | Log2 Fold-change | *P*-value | Log2 Fold-change | *P*-value | Log2 Fold-change | *P*-value | Log2 Fold-change | *P*-value |
| M model (29) | | | | | | | | | | | |
| TC17341 | actin, clone 205-like (LOC551369), | XM_003251415.1 | Hsp70 family protein | 2.440979 | 2.9E-06 | 2.854189 | 1.59E-08 | 3.529624 | 4.77E-16 | 2.953796 | 1.33E-10 |
| DB752873 | titin | EFN83272.1 | - | 2.423906 | 2.74E-08 | 2.95105 | 9.92E-13 | 3.102513 | 3.19E-15 | 2.647332 | 6.58E-11 |
| NP9552724 | LOC410736 | XM_394212.3 | F36G9.12 | 2.388677 | 4.86E-14 | 2.393438 | 5.77E-13 | 3.446603 | 3.41E-38 | 3.416794 | 3.59E-41 |
| DB731917 | Troponin T | DB731917 | Troponin T isoform 2 | 2.383264 | 3E-05 | 2.661544 | 2.63E-06 | 3.395872 | 1.96E-12 | 3.099568 | 1.55E-10 |
| TC14607 | sn1-specific diacylglycerol lipase beta-like (LOC726798), | XM_001122519.2 | AGAP012171-PA | 1.798301 | 7.02E-07 | 2.154584 | 1.54E-09 | 2.211448 | 1.44E-10 | 1.687442 | 2.31E-06 |
| TC22963 | myosin heavy chain 1, transcript variant 1 (Mhc1) | XM_393334.4 | PREDICTED: similar to Myosin heavy chain CG17927-PB isoform B isoform 1 | 1.714125 | 7.61E-14 | 1.862874 | 2.17E-15 | 2.841322 | 9.51E-50 | 2.199787 | 2.36E-27 |
| TC24102 | troponin I | NM_001040256.1 | Troponin I isoform 6a1 | 1.631501 | 9.87E-10 | 1.719888 | 4.98E-10 | 3.059444 | 1.26E-47 | 3.050012 | 1.65E-52 |
| TC14861 | hypothetical protein LOC409060 | XM_397543.4 | Chromosome undetermined scaffold_61 | 1.630357 | 7.75E-17 | 2.031493 | 2.70E-26 | 3.477002 | 1.80E-130 | 3.193686 | 9.13E-111 |
| BI504328 | - | - | unknown | 1.598277 | 8.65E-07 | 1.634577 | 1.47E-06 | 3.169868 | 7.04E-37 | 2.710525 | 5.64E-26 |
| TC13807 | Tropomyosin-1 | EFN78348.1 | Tropomyosin-1 | 1.581714 | 3.96E-62 | 1.478633 | 1.98E-47 | 2.902855 | 0 | 2.638584 | 2.14E-263 |
| DB730255 | - | - | Putative uncharacterized protein PY01729 | 1.568047 | 3.82E-21 | 1.922253 | 1.14E-31 | 3.039829 | 4.76E-120 | 2.769777 | 2.08E-101 |
| TC15188 | Myosin regulatory light chain 2 | EFN88243.1 | Myosin light chain | 1.551054 | 4.94E-68 | 1.618083 | 3.60E-68 | 2.819843 | 0 | 2.562358 | 6.54E-279 |
| TC14580 | paramyosin | XP_002432355.1 | Paramyosin, long form | 1.548532 | 6E-138 | 2.011497 | 3.56E-246 | 2.946677 | 0 | 2.862466 | 0 |
| TC22830 | Titin | EFN80687.1 | PREDICTED: similar to CG32019-PA isoform A | 1.533425 | 8.69E-13 | 1.695491 | 1.11E-14 | 2.777551 | 2.59E-56 | 2.03833 | 4.68E-27 |
| TC12842 | Muscle-specific protein 20 | EFN86046.1 | Calponin/transgelin | 1.494772 | 1.5E-10 | 2.210437 | 1.20E-24 | 3.664905 | 4.13E-115 | 3.485626 | 2.04E-108 |
| NP1608872 | troponin T | NM_001040258.1 | troponin T | 1.467533 | 9.79E-70 | 1.484005 | 1.57E-64 | 2.5793 | 2.09E-298 | 2.139864 | 2.17E-198 |
| TC15753 | PDZ and LIM domain protein 2 | EFN88596.1 | IP16036p | 1.457853 | 1.19E-07 | 1.428425 | 9.61E-07 | 2.928795 | 8.53E-42 | 2.587669 | 1.15E-32 |
| TC15638 | fau protein | 218505757 | Anoxia | 1.432173 | 2.49E-06 | 2.540528 | 2.40E-22 | 3.875602 | 1.08E-84 | 3.755996 | 2.13E-84 |
| TC18912 | troponin T (TpnT) gene, complete cds, alternatively spliced | BK005281.1 | ABC-type spermidine/putrescine transport system, ATPase component | 1.423906 | 1.1E-10 | 1.438179 | 5.74E-10 | 2.31717 | 3.08E-33 | 2.058449 | 2.82E-27 |
| NP9550064 | mlck | EFN64027.1 | Stretchin-Mlck CG18255-PA, isoform A | 1.406851 | 1.36E-89 | 2.279975 | 2.43E-286 | 2.948557 | 0 | 2.640532 | 0 |
| TC14294 | Zeelin1 | 110759392 | PREDICTED: similar to Zeelin1 CG6803-PD, isoform D | 1.378102 | 7.65E-14 | 1.417618 | 2.11E-13 | 2.939863 | 1.77E-94 | 2.044767 | 2.69E-38 |
| TC12464 | β-actin | 256862184 | Actin, clone 205, transcript variant 1 (LOC551369) | 1.30728 | 4.34E-45 | 1.246341 | 2.91E-36 | 2.398193 | 7.51E-206 | 1.735198 | 2.75E-97 |
| DB737629 | titin | EFN83272.1 | BMKETTIN | 1.300802 | 1.1E-08 | 2.111771 | 1.99E-24 | 2.701032 | 4.05E-50 | 2.405671 | 1.76E-40 |
| BP540149 | mrjp2 | 58585108 | Major royal jelly protein 2 precursor | 1.257206 | 2.01E-49 | 2.401212 | 3.27E-238 | 2.285331 | 4.70E-217 | 1.512524 | 3.97E-83 |
| TC14544 | regucalcin | EFN67386.1 | PREDICTED: similar to Senescence marker protein-30 CG7390-PA isoform A | 1.19325 | 3.8E-82 | 1.840642 | 2.83E-218 | 1.984144 | 2.52E-280 | 1.701416 | 4.46E-212 |
| TC13222 | mrjp7 | 62198227 | major royal jelly protein 7 | 1.123706 | 4.8E-286 | 2.805433 | 0 | 2.943341 | 0 | 1.475218 | 0 |
| TC20289 | lim | 66514673 | Muscle LIM protein at 84B CG1019-PA, isoform A isoform 1 | 1.123397 | 8.81E-09 | 1.581256 | 8.02E-17 | 2.881299 | 9.73E-87 | 2.518635 | 1.21E-65 |
| TC12457 | CG1674-PD | 110766214 | PREDICTED: similar to CG1674-PD, isoform D | 1.111962 | 1.63E-05 | 1.169691 | 1.41E-05 | 2.08353 | 8.09E-21 | 1.601142 | 2.22E-12 |
| TC24109 | mrjp2 | 58585108 | major royal jelly protein 2 precursor | 1.09413 | 0 | 2.298315 | 0 | 2.348336 | 0 | 1.247057 | 0 |
| R model (24) | | | | | | | | | | | |
| TC14045 | smcp1a | EFN88815.1 | Structural maintenance of chromosomes protein 1A | -2.22403 | 7.54E-78 | -5.91565 | 4.85E-144 | -7.07029 | 4.18E-164 | -6.33417 | 8.14E-198 |
| DB752140 | igr | XR_120226 | PREDICTED: Apis mellifera ionotropic glutamate receptor (LOC411220), miscRNA | -1.54808 | 2.75E-07 | -2.74785 | 8.74E-12 | -3.48745 | 1.92E-15 | -2.92126 | 4.64E-16 |
| TC16687 | hdcp | EFN61335.1 | Hexosaminidase domain-containing protein | -1.29432 | 7.02E-06 | -1.52545 | 2.51E-06 | -1.75048 | 7.24E-08 | -1.25829 | 3.46E-06 |
| BI509901 | IGFn3-10 | XM_003251322 | immunoglobulin-like and fibronectin type III domain containing 10 | -1.27209 | 5.59E-07 | -1.35638 | 1.08E-06 | -2.61055 | 6.15E-15 | -2.09683 | 4.08E-14 |
| TC13885 | prohormone-1 | XM_001121443 | PREDICTED: Apis mellifera prohormone-1 (LOC725616) | -1.26655 | 2.34E-05 | -1.70632 | 1.07E-06 | -1.7785 | 1.88E-07 | -1.68179 | 3.35E-08 |
| TC21840 | Sodium/potassium-transporting ATPase subunit alpha | EFN66583.1 | Sodium pump alpha subunit | -1.24248 | 1.92E-05 | -2.16549 | 4.89E-09 | -1.85065 | 3.99E-08 | -1.69949 | 1.16E-08 |
| TC15755 | Regulator of G-protein signaling 20 | EFN86245.1 | AGAP007127-PA | -1.22246 | 3.11E-07 | -1.89171 | 7.17E-11 | -2.11048 | 3.89E-13 | -1.64617 | 1.40E-11 |
| TC21748 | Gelsolin precursor | XM_003697981.1 | Gelsolin precursor (Actin-depolymerizing factor) (ADF) (Brevin) (AGEL) | -1.221 | 1.57E-05 | -1.54233 | 1.81E-06 | -2.76736 | 7.68E-13 | -1.76821 | 1.86E-09 |
| NP9553100 | LOC550984 | XM_623379.2 | hypothetical protein LOC550984 | -1.21038 | 4.29E-07 | -1.54522 | 1.70E-08 | -1.97288 | 4.42E-12 | -1.70625 | 5.07E-12 |
| BI515609 | - | - | ComG operon protein 2 | -1.20752 | 3.45E-08 | -1.70393 | 3.79E-11 | -1.90588 | 1.35E-13 | -1.75472 | 1.41E-14 |
| TC15827 | ionotropic glutamate receptor (LOC411220) | XP_316066.4 | AGAP006027-PA | -1.18473 | 4.29E-17 | -2.3243 | 6.28E-36 | -4.74197 | 5.32E-71 | -3.74282 | 1.87E-74 |
| TC22739 | Phosphatase and actin regulator 1 | EFN72033.1 | AGAP006812-PA | -1.16409 | 3.66E-12 | -1.17438 | 7.61E-11 | -1.34839 | 6.36E-14 | -1.09683 | 2.99E-12 |
| TC17621 | Poly(rC)-binding protein 3 | EFN79734.1 | - | -1.14846 | 2.08E-06 | -1.34127 | 6.71E-07 | -2.68856 | 3.72E-16 | -1.80044 | 2.30E-12 |
| TC23120 | - | - | Putative uncharacterized | -1.11476 | 3.22E-05 | -1.27706 | 1.78E-05 | -1.27969 | 8.32E-06 | -1.22807 | 2.15E-06 |
| BE844531 | sodium/potassium-transporting ATPase subunit beta-2-like (LOC408722) | XM_392257.4 | Nodule-specific glycine-rich protein 2B | -1.11342 | 2.55E-13 | -1.92512 | 2.63E-24 | -1.85348 | 8.26E-25 | -1.67163 | 7.53E-26 |
| NP9548518 | low density lipoprotein receptor-related protein-like protein | NP_001116809.1 | CG32432-PA | -1.11215 | 3.07E-06 | -1.8428 | 3.08E-10 | -1.61411 | 2.55E-09 | -1.49538 | 5.91E-10 |
| BI510721 | PREDICTED: hypothetical protein | XP_001120038.1 | Hepatoma-derived growth factor-related protein 2 | -1.10966 | 1.75E-09 | -2.01338 | 5.89E-18 | -2.34859 | 6.61E-23 | -2.16572 | 4.91E-25 |
| TC19664 | hypothetical LOC100577066 | XR_120142.1 | Chromosome undetermined scaffold_118, whole genome shotgun sequence | -1.10635 |  | -1.52944 | 1.26E-13 | -1.7418 |  | -1.70285 | 8.58E-20 |
| TC21571 | Matrix metalloproteinase-17 | EFN85109.1 | CG1794-PA isoform A | -1.10623 | 2.68E-07 | -2.1987 | 5.63E-15 | -2.16891 | 8.88E-16 | -2.03712 | 1.67E-17 |
| TC17711 | AGAP000822-PA | XP_316786.3 | PREDICTED: hypothetical protein | -1.07045 | 1.28E-05 | -1.09334 | 3.77E-05 | -2.16998 | 2.52E-12 | -1.1238 | 1.70E-06 |
| NP9544343 | Protein still life, isoforms C/SIF type 2 | EFN76027.1 | still life CG5406-PC, isoform C (LOC724526), | -1.04699 | 1.9E-16 | -1.7247 | 5.72E-29 | -2.58829 | 1.84E-50 | -2.19898 | 1.63E-50 |
| DB730137 | hypothetical LOC100749243 | XR_135619.1 | Peptidyl-prolyl cis-trans isomerase | -1.01747 | 8.53E-11 | -1.76472 | 5.24E-20 | -1.5245 | 1.14E-17 | -1.33987 | 1.68E-17 |
| TC13737 | Rho GTPase-activating protein 100F | EFN67989.1 | AGAP003944-PA | -1.00905 | 3.21E-05 | -1.21887 | 7.08E-06 | -2.41791 | 7.97E-14 | -1.15128 | 1.06E-06 |
| TC15513 | ABC transporter G family member 20 | EFN84917.1 | AGAP002638-PA | 1.245405 | 1.96E-08 | 1.076581 | 8.08E-06 | 1.220464 | 9.92E-08 | 2.580465 | 1.07E-52 |

**Table S7** Top 20 of GO Enrichment analysis in samples (p≤0.05)

| # | Gene Ontology term | =-LOG10(PVALUE) | ontology | groups |
| --- | --- | --- | --- | --- |
| 1 | structural molecule activity | 23.38615818 | MF | Sample 5 VS 1 |
| 2 | membrane | 13.12205305 | CC | Sample 4 VS 1 |
| 3 | membrane | 13.10846254 | CC | Sample 3 VS 1 |
| 4 | signaling | 12.60380065 | BP | Sample 4 VS 1 |
| 5 | cell periphery | 12.42945706 | CC | Sample 4 VS 1 |
| 6 | plasma membrane | 11.67778071 | CC | Sample 4 VS 1 |
| 7 | plasma membrane part | 11.40120949 | CC | Sample 4 VS 1 |
| 8 | intrinsic to membrane | 11.1266794 | CC | Sample 3 VS 1 |
| 9 | membrane part | 10.41907502 | CC | Sample 3 VS 1 |
| 10 | contractile fiber part | 9.200659451 | CC | Sample 2 VS 1 |
| 11 | contractile fiber | 9.00656377 | CC | Sample 2 VS 1 |
| 12 | anatomical structure development | 8.924453039 | BP | Sample 4 VS 1 |
| 13 | actin cytoskeleton | 8.614393726 | CC | Sample 2 VS 1 |
| 14 | myosin complex | 8.492144128 | CC | Sample 2 VS 1 |
| 15 | membrane part | 7.899629455 | CC | Sample 4 VS 1 |
| 16 | sarcomere | 7.782516056 | CC | Sample 2 VS 1 |
| 17 | plasma membrane | 7.655607726 | CC | Sample 3 VS 1 |
| 18 | myofibril | 7.607303047 | CC | Sample 2 VS 1 |
| 19 | system development | 7.496209317 | BP | Sample 4 VS 1 |
| 20 | cell periphery | 7.244887734 | CC | Sample 3 VS 1 |

**Table S8** The most abundant differentially expressed signalling pathways shared in the samples except sample 1 (Top 5 in bold).

| Pathway | Pathway ID | Groups | DEGs(annotated) | Qvalue |
| --- | --- | --- | --- | --- |
| **Ribosome** | ko03010 | 5_3 (332) | 72 (21.69%) | 9.28E-68 |
|  |  | 5_2 (488) | 69 (14.14%) | 8.03E-51 |
|  |  | 5_1 (940) | 58 (6.17%) | 2.57E-21 |
|  |  | 5_4 (401) | 40 (9.98%) | 3.82E-21 |
| **Phosphatidylinositol signaling system** | ko04070 | 4_1 (1322) | 44 (3.33%) | 5.48E-07 |
|  |  | 4_2 (789) | 29 (3.68%) | 6.20E-05 |
|  |  | 3_1 (566) | 17 (3%) | 0.03857639 |
|  |  | 4_3 (263) | 12 (4.56%) | 0.01819079 |
| **Phototransduction - fly** | ko04745 | 4_1 | 30 (2.27%) | 2.83E-05 |
|  |  | 4_2 | 20 (1.51%) | 7.55E-04 |
|  |  | 5_1 | 19 (2.02%) | 1.24E-02 |
|  |  | 3_1 | 15 (2.65%) | 0.01094424 |
|  |  | 4_3 | 8 (3.04%) | 0.02902571 |
| **Drug metabolism - cytochrome P450** | ko00982 | 5_1 | 20 (2.13%) | 7.54E-03 |
|  |  | 5_2 | 16 (3.28%) | 1.47E-04 |
|  |  | 5_3 | 14 (4.22%) | 2.98E-05 |
|  |  | 4_3 | 9 (3.42%) | 0.01819079 |
| **Metabolism of xenobiotics by cytochrome P450** | ko00980 | 5_1 | 20 (2.13%) | 1.24E-02 |
|  |  | 5_2 | 16 (3.28%) | 3.05E-04 |
|  |  | 5_3 | 14 (4.22%) | 5.77E-05 |
|  |  | 4_3 | 9 (3.42%) | 0.01863344 |
| Drug metabolism - other enzymes | ko00983 | 5_1 | 19 (2.02%) | 2.29E-02 |
|  |  | 5_2 | 17 (3.48%) | 1.18E-04 |
|  |  | 5_3 | 16 (4.82%) | 4.32E-06 |
|  |  | 4_3 | 8 (3.04%) | 0.03673858 |
| ECM-receptor interaction | ko04512 | 4_1 | 19 (1.44%) | 2.07E-02 |
|  |  | 4_2 | 16 (2.03%) | 1.51E-03 |
|  |  | 3_1 | 12 (2.12%) | 0.01351374 |
|  |  | 5_2 | 10 (2.05%) | 1.59E-02 |
|  |  | 3_2 (90) | 6 (6.67%) | 0.00126263 |
| Natural killer cell mediated cytotoxicity | ko04650 | 4_1 | 19 (1.44%) | 6.05E-03 |
|  |  | 3_1 | 10 (1.77%) | 0.03857639 |
| Retinol metabolism | ko00830 | 5_1 | 18 (1.91%) | 7.34E-03 |
|  |  | 5_2 | 15 (3.07%) | 1.11E-04 |
|  |  | 5_3 | 13 (3.92%) | 2.40E-05 |
|  |  | 4_3 | 8 (3.04%) | 0.01819079 |
| Linoleic acid metabolism | ko00591 | 5_1 | 14 (1.49%) | 1.91E-02 |
|  |  | 5_2 | 11 (2.25%) | 2.02E-03 |
|  |  | 4_3 | 6 (2.28%) | 0.03947017 |
|  |  | 5_3 | 10 (3.01%) | 3.39E-04 |
| Starch and sucrose metabolism | ko00500 | 5_2 | 11 (2.25%) | 4.58E-03 |
|  |  | 5_3 | 8 (2.41%) | 1.84E-02 |
|  |  | 4_3 | 7 (2.66%) | 0.02655423 |
| RNA polymerase | ko03020 | 4_3 | 5 (1.9%) | 0.03059806 |
| Other glycan degradation | ko00511 | 2_1 (91) | 4 (4.4%) | 0.00531324 |
